# Daily life in the Open Biologist’s second job, as a Data Curator

**DOI:** 10.12688/wellcomeopenres.22899.1

**Published:** 2024-09-12

**Authors:** Livia C.T. Scorza, Tomasz Zieliński, Irina Kalita, Alessia Lepore, Meriem El Karoui, Andrew J. Millar

**Affiliations:** 1 Centre for Engineering Biology and School of Biological Sciences, University of Edinburgh, Edinburgh, Scotland, EH9 3BF, UK; 2 Institute of Cell Biology, School of Biological Sciences, University of Edinburgh, Edinburgh, Scotland, EH9 3JD, UK; 3 Center for Synthetic Microbiology (SYNMIKRO), Max Planck Institute for Terrestrial Microbiology, Marburg, Germany; 4 Laboratory for Optics and Biosciences, École Polytechnique, Institut Polytechnique de Paris, Palaiseau, Île-de-France, France; 5 Laboratoire de Biologie et Pharmacologie Appliquée (LBPA), - ENS Paris-Saclay CNRS UMR 8113, Paris, Gif-sur-Yvette, France

**Keywords:** Open science, FAIR, data sharing, data curation, repositories, reproducibility, accessibility, biological data, datasets, metadata.

## Abstract

**Background:**

Data reusability is the driving force of the research data life cycle. However, implementing strategies to generate reusable data from the data creation to the sharing stages is still a significant challenge. Even when datasets supporting a study are publicly shared, the outputs are often incomplete and/or not reusable. The FAIR (Findable, Accessible, Interoperable, Reusable) principles were published as a general guidance to promote data reusability in research, but the practical implementation of FAIR principles in research groups is still falling behind. In biology, the lack of standard practices for a large diversity of data types, data storage and preservation issues, and the lack of familiarity among researchers are some of the main impeding factors to achieve FAIR data. Past literature describes biological curation from the perspective of data resources that aggregate data, often from publications.

**Methods:**

Our team works alongside data-generating, experimental researchers so our perspective aligns with publication authors rather than aggregators. We detail the processes for organizing datasets for publication, showcasing practical examples from data curation to data sharing. We also recommend strategies, tools and web resources to maximize data reusability, while maintaining research productivity.

**Conclusion:**

We propose a simple approach to address research data management challenges for experimentalists, designed to promote FAIR data sharing. This strategy not only simplifies data management, but also enhances data visibility, recognition and impact, ultimately benefiting the entire scientific community.

## Plain language summary

Researchers should openly share data associated with their publications unless there is a valid reason not to. Additionally, datasets have to be described with enough detail to ensure that they are reproducible and reusable by others. Since most research institutions offer limited professional support in this area, the responsibility for data sharing largely falls to researchers themselves. However, many research groups still struggle to follow data reusability principles in practice. In this work, we describe our data curation (data organization and management) efforts working directly with the researchers who create the data.

We show the steps we took to organize, standardize, and share several datasets in biological sciences, pointing out the main challenges we faced. Finally, we suggest simple and practical data management actions, as well as tools that experimentalists can integrate into their daily work, to make sharing data easier and more effective.

## Disclaimer

The views expressed in this article are those of the authors. Publication in Wellcome Open Research does not imply endorsement by Wellcome.

## Introduction

Scientific progress relies not only on the production of good quality data that ultimately leads to the publication of peer reviewed articles, but also on the re-analysis of the underlying datasets, which requires their public release. Sharing datasets is the bedrock of research data transparency, reproducibility and reusability. While it discourages fraudulent data manipulation and can reduce research duplication and hence costs, it also brings other benefits for users and data owners, such as fostering collaborations, increasing impact and generating new types of research output ^1, 2^. Data sharing is not only good research practice, but it has become a requirement for many datasets funded by most funding bodies (e.g., UKRI, Wellcome, Royal Society, EMBO). Additionally, many peer-reviewed journals now have data sharing policies that require the inclusion of a “data availability statement” where authors must add the links to repository records that hold the datasets supporting the results presented.

Data openness, however, does not automatically guarantee data reusability. To address reproducibility and reusability issues, the FAIR (Findable, Accessible, Interoperable, Reusable) principles were published as a general guidance to share research data ^3^. According to these principles, (meta)data must be findable, i.e., datasets are assigned with persistent identifiers such as a digital object identifier (DOI), indexed in a searchable resource and described with rich metadata. Users must be able to access and download the meta(data) using standardised communications protocols such as http(s). Datasets should be also easily interpreted by computers without specialized algorithms or vendor specific software, and metadata written using standard, subject-specific vocabularies and linked to other metadata resources when possible (interoperable). Reusability is achieved when the other principles are implemented, and when meta(data) contains all relevant details for data reproducibility and a data usage license is provided to clarify the conditions for reuse.

**Figure 1.**
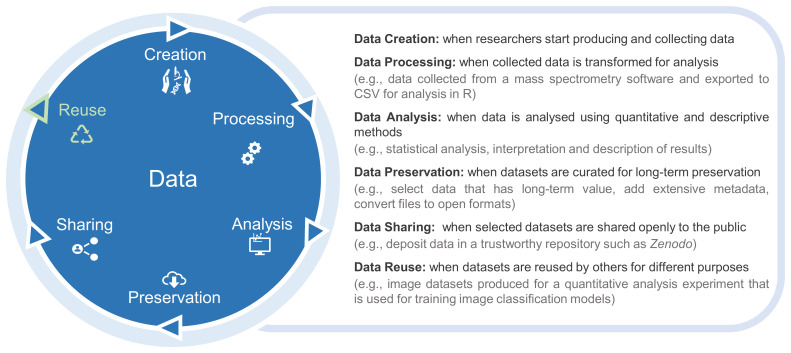
The stages of the research data life cycle are depicted in the blue circle, with each stage explained on the right.

As the scientific community acknowledged the importance of open and reusable data, several tools to facilitate data sharing and reproducibility have been developed. There is a large landscape of online repositories for sharing data, as well as tools for producing consistent metadata either using specific software or following minimum information standards established for different disciplines ^4– 12^. However, there is still a gap between the reality of sharing practices and what is mandated by Open and FAIR principles. Among the perceived factors stopping researchers from sharing data are the time and effort to prepare data in quality suitable for sharing, as datasets need to be formatted and sufficiently described to achieve FAIR standards, as well as the lack of support and practical knowledge in effective research data curation and organization ^13– 17^. Even when researchers claimed that they shared their data, it has been observed that datasets were frequently not reusable because essential metadata information was missing, or because datasets were incomplete or no longer available ^18– 20^. Although various resources now point researchers towards open science tools and practices, the literature lack practical guidance for preparing FAIR data from the perspective of experimental researchers, and primarily addresses the curation of biological data from the viewpoints of database managers and data aggregators ^21– 23^. One of the missions of our group, the Biological Research Data Management (BioRDM) team, is to make data sharing a simpler task for experimental researchers in biological sciences by integrating data sharing among the routine tasks across the entire research data life cycle ( Figure 1). As the driving force of the data life cycle is data reuse, researchers must aim to have FAIR data ready at the sharing stage. However, datasets frequently require some degree of data curation beyond researchers’ normal practice to make them FAIR. Our team works collaboratively with research groups generating data, where we help them to comply with FAIR principles by curating datasets for public sharing. In this article we show the rationale behind our data curation process, explaining how we selected, organized and shared datasets, and which tools and data management tasks were applied. We highlight the main data problems and difficulties we encountered and how we resolved them, as well as the positive elements found. Finally, we consider how to implement data management practices throughout the research data life cycle to facilitate collaboration and sharing. We show that sharing does not need to be an all or nothing approach and researchers can choose the steps and practices that most align with their data type and experimental context. It is relevant to note that we are only dealing with open data and not handling datasets with sharing restrictions.

## Data curation

Throughout the text we often showcase three datasets that were shared with the support of the BioRDM team. The datasets were shared on Zenodo, a generalist data repository funded by CERN and OpenAire ( https://zenodo.org/), within the BioRDM community on that resource ( https://zenodo.org/communities/biordm/):

- “SARS-CoV-2 RNA levels in Scotland’s wastewater” ^24, 25^, herein presented as Scorza2022;
- “An Hfq-dependent post-transcriptional mechanism fine tunes RecB expression in *Escherichia coli*” ^26, 27^, herein presented as Kalita2023;
- “In vivo single-molecule imaging of RecB reveals efficient repair of DNA damage in *Escherichia coli*” ^28, 29^ herein presented as Lepore2024.

All the datasets were released as data supporting publications. Scorza2022 was a collaborative project involving different institutions developing methods for detection of SARS-CoV-2 in wastewater, as well as data analysis strategies, as part of COVID-19 monitoring programme in Scotland ^25^. Our work was to select the most appropriate data and other useful outputs for sharing. Because of the collaborative nature of this project, the dataset presented data and protocol integration challenges, which will be exemplified here. Kalita2023 and Lepore2024 reflect data curation alongside a research group, while preparing manuscripts to publish. Their work focuses on understanding DNA repair mechanisms in the model bacterial organism *Escherichia coli* by using data from single molecule microscopy in different strains and environmental conditions. The curation for these datasets had less data integration challenges, but still required several data organizational and annotation steps.

**Figure 2.**
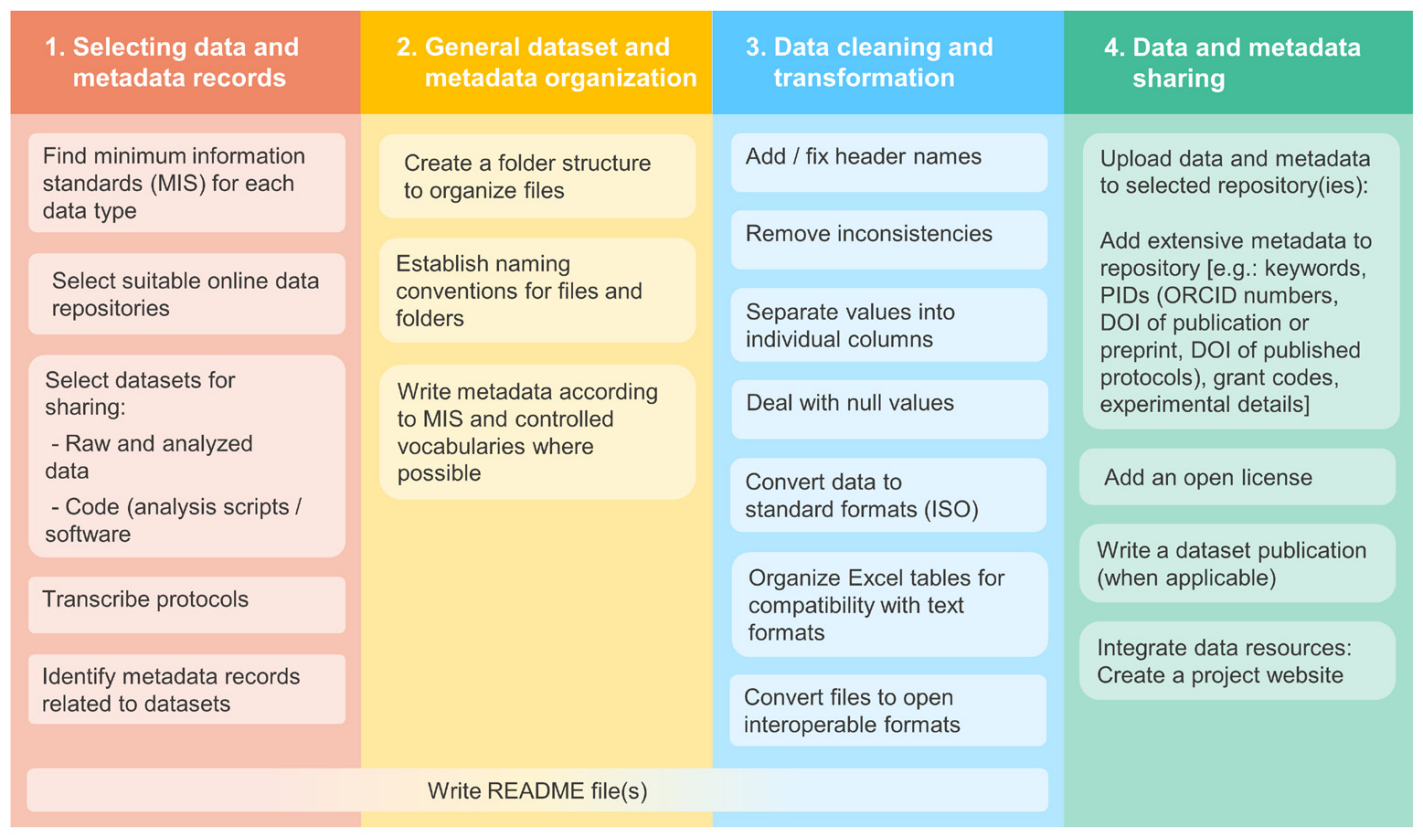
The data curation steps performed by our team while preparing (FAIR) data for sharing.

The main steps of our data curation methodology are described below and summarized in Figure 2.

### Selecting data and metadata records for sharing

The main investigators and data producers should collaboratively determine which data outputs from each project hold potential interest for the scientific community, thus warranting sharing. For research data accompanying a publication, all data used to produce figures and supporting conclusions should normally be shared. This can include raw data (e.g., images used to extract numerical data, raw RT-qPCR data, chromatograms, raw sequencing data, etc.) as well as processed data (e.g., normalized data, image segmentation files). The dataset must also include any original or modified methodologies to produce and analyse the data, such as protocols, hardware designs and code.

Selecting exactly which data files and information to include when sharing a dataset will depend on the type of data. For some data types data in biological sciences, there are "minimum information" guidelines available, known as “minimum information standards (MIS)”. Prior to selecting the (meta)data files, we check if MIS are available for each data type. We also search for suitable data specific repositories, as the file formats and metadata required by repositories can be used as guidance when selecting and organising (meta)data. Trustworthy repositories and MIS can be found in FAIRsharing ( https://fairsharing.org/) and re3data ( https://www.re3data.org/. More specifically, the Minimum Information for Biological and Biomedical Investigations (MIBBI) contains a collection of the most known standards in biology ( https://fairsharing.org/3518). Funders and journals also have their own lists of recommended repositories.

**Figure 3.**
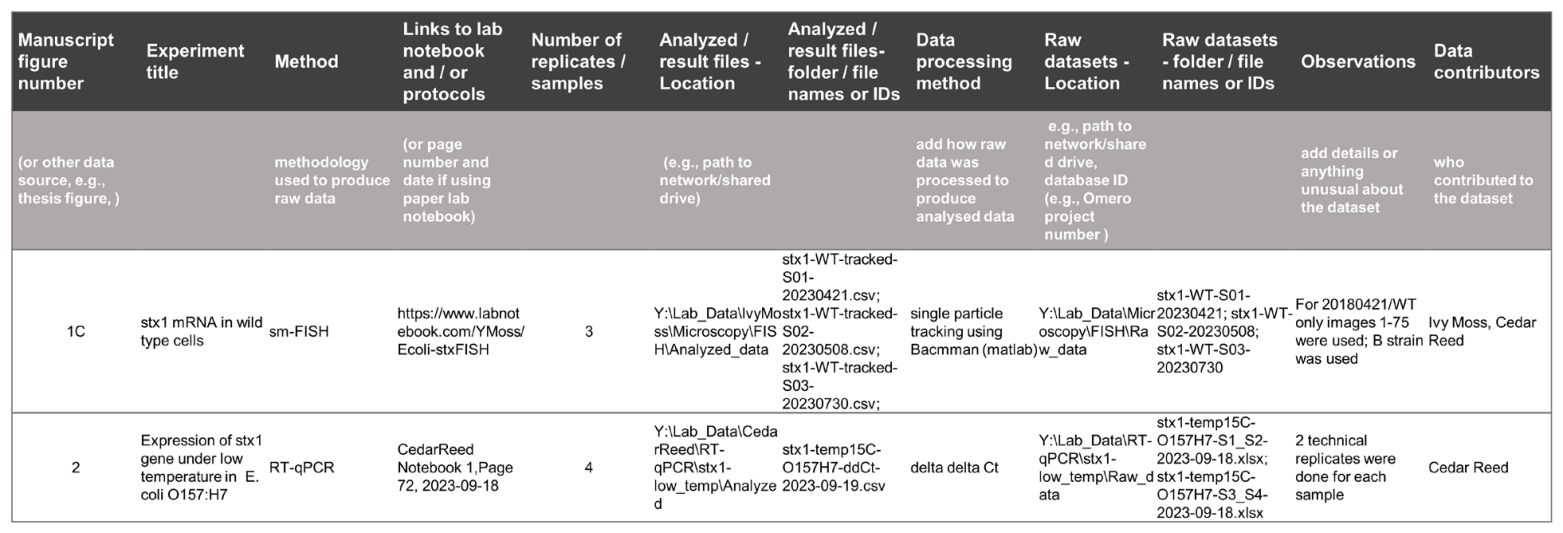
A template for a spreadsheet to be used while preparing figures for a publication with example entries. Researchers should log their manuscript figure numbers and all the background information about the data used to produce each figure, such as: experiment title, links for metadata information (e.g., laboratory notebook entries), the locations of the datasets in their network drive, folders and file names containing the data, observations and contributors. Such a table can help anyone to quickly trace back to the raw and analysed datasets used in a publication, making it easier to select files for sharing.

***Raw and analysed data.*** The main challenge for selecting data was finding the correct data files related to the manuscript figures. It was essential that we could be in contact with the authors to clarify the location and names of raw and analysed data files supporting the results being presented. To help us retrieve the correct files, we prepared a simple spreadsheet that authors filled adding data location and links to dataset description such as links to electronic laboratory notebooks entries ( Figure 3 and “template_for_source_data_information.xlsx” on Zenodo ^30^). The idea is that this spreadsheet is also used when preparing figures for manuscripts or theses, as it will help to keep track of original raw data, facilitating data preservation and sharing stages.

In the Scorza2022 dataset we dealt with SARS-CoV-2 viral genetic material quantified by RT-qPCR in wastewater samples across Scotland. The samples were usually from the inflow to wastewater treatment plants. The values of viral gene copies per litre were reported for all replicates in all locations measured, together with the mean values. Furthermore, the population-normalised values, reported as million gene copies per person per day, were shared for all replicates. Parameters used to produce normalised values were also included, such as incoming flow and ammonia levels, as well as other accompanying variables, such the dates, geographical locations of sample collection sites and population size. Additionally, all methods to produce the data was shared (see below in “Protocols”) aiming at reusability by other institutions interested in wastewater-based epidemiology monitoring.

For the Kalita2023 and Lepore2024 datasets, data from labelled mRNAs and proteins in *E. coli* cells were presented in graphs, which were derived from the analysis of fluorescence microscopy images. In this case, our approach was to select all the raw data and the methodology used to obtain the values presented. That included raw image and video data, the scripts used for processing images and obtaining the numerical values, the derived numerical values, and processed images (masks of segmented cells). The raw images/videos and segmentation masks were shared with the aim of transparency and validation so the data can be verifiable, and also for training purposes, in case one wishes to re-run the image analysis scripts and have results to compare with. The raw images can also be reused as a control dataset for those testing different algorithms for detection of fluorescent spots in cell crowded environments, as well as testing algorithms for automated cell segmentation. For the derived numerical values used in the quantitative analysis, all numbers for all replicates were included, and not only the averages. Kalita2023 also presented gene expression analysis from quantitative real-time PCR (RT-qPCR), and the same rule of sharing values for all replicates and not only the averages shown in the graphs was applied. We also followed the MIS for RT-qPCR experiments, MIQE, as closely as possible ^31^, sharing raw Ct values, normalized data and fold changes, as well as all the experimental details (metadata and protocols).

***Protocols.*** Original and modified protocols were identified for sharing together with datasets. Scorza2022 involved multiple institutions and obtaining the correct, up to date protocols was more challenging, as the methodologies were scattered in different locations, such as institution’s websites, reports or internal laboratory files. Discrepancies between different versions of the protocols were also an issue to be resolved *.* Communication with the authors was key to confirm and update protocols that had been modified since their first version. We transcribed the most up to date protocols on a protocol sharing platform, *protocols.io* ^32– 34^. ( https://www.protocols.io/) *Protocols.io* allows users to share the protocol with collaborators, so the protocols could be checked and modified where necessary by the different members of the project before publishing. If updates are needed after publishing the protocol, the updated version can be published whilst keeping the previous versions and the same DOI.

***Code (analysis scripts and software).*** Any code developed to produce and/or analyse a dataset, should be shared with an open-source license. In our data curation work the issues we found when sharing code were mostly related to code that had been shared without a licence. For the groups we worked with we advised on suitable open licenses to add. When reusing code from others that did not have a clear license, we first clarified with the code creators which license they would like to add to their code so we could cite and re-share their software package appropriately.

Ideally code use should not be dependent on commercial software. However, we are aware that certain commercial programs such as MATLAB ( https://mathworks.com) are widely used in biological sciences workflows, such as image analysis or bioinformatics, and some MATLAB files can be used in open-source software such as Octave ^35^ https://octave.org/. When following the best possible open-source practices, MATLAB code can still be shared publicly. Analysis scripts from Kalita2023 and Lepore2024 were shared in MATLAB format on Zenodo together with the full dataset, and the dataset was linked to their corresponding GitLab pages. Additional to the code, documentation containing scripts usage and instructions was provided in specific README or analysis workflow text files. Controlled test datasets and parameters were also included to facilitate testing, validation and reuse.

***Metadata.*** Metadata is the description of the data, and it is particularly important for data reproducibility. In order to prepare fully descriptive metadata to share with data files, we identified information searching in different sources from the data creators, such as records in electronic laboratory notebooks (ELNs), information written in spreadsheets together with data files, metadata files generated automatically by software (e.g., microscopy image parameters saved during image acquisition), and descriptions in reports and in the manuscripts prepared for publication. Particularly, we found that having access to ELNs facilitated the accessibility and reusability of metadata, as experimental details could be easily copied and edited for clarification in our metadata files. Again, communication with data creators was key to clarify ambiguities and cases where samples were poorly described. From these sources, we were able to create descriptive metadata in the form of README files to accompany the datasets. We explain how we wrote README files in “Writing Metadata: README files” section.

### General dataset and metadata organization

After identifying and selecting the datasets to share, we had a good understanding of the distinct data types being presented, as well as the number of files for each data type and their categories (e.g., raw sequencing or imaging data, analysed data, data tables, etc). This allowed us to prepare an organizational structure for the files in folders, as well as organize metadata files. These steps will be described below.

***Creating a folder structure to organize files.*** The aim of organizing data in a sensible folder structure is to improve data findabilty by interested users. The folder structure used to organize the data will depend on the type of research, the number of files and parameters, or even on the stage of the research project. Some scientific fields already have established standards for organizing data, such as the “Brain Imaging Data Structure” ^36^ or computing science projects ^37^. Following the same standard when organizing similar data types optimizes data reuse, as users will intuitively search and interact with the data when they already know the structure, and the same analysis pipelines can be used for different datasets. Research groups can also create their own structure based on their data user audience and how they expect others to reuse the data. For example, is it more likely that they will be interested in the raw datasets or in the analysis pipeline? Is it expected that they will reanalyse your data or just reuse your code for their own data?

When sharing data presented in a publication, a folder structure that follows the order of the figures presented in the paper can be a good option, as it will help readers to quickly find the related data files. For Kalita2023, we followed this structure first dividing the dataset in main data types (Growth and viability data, Fluorescence microscopy, Source code and RT-qPCR) and then organizing the data types by figure numbers of the publication (e.g., folders “Fig1-RecB_mRNAs_abundance; " Fig2-RecB_protein_abundance"). Each figure folder had its files split into raw data and quantification results. However, for Lepore2024, we chose to organize data “by genotype” instead of “by figure”, because Lepore2024 used the same image datasets to produce different figures in the manuscript. A text document showing the relationship between datasets and figures was included to help readers to quickly find the correct data. Image analysis scripts were included in a separate folder.

Scorza2022 was a “data descriptor” type of publication and its figures were mainly for illustrational purposes, so the dataset was divided in data types. In this case most files were numerical data in CSV format, but the dataset also contained processing scripts. The files were then split in a “data” folder and a source code folder named “src”. The “data” folder contained the CSV files representing the values for quantification of the N1 gene of SARS-CoV-2. “src” contained R files with scripts used for data transformation, as well as scripts used to produce the interactive map and heatmap figures shown in the publication.

***Establishing naming conventions for files and folders.*** In addition to having a thoughtful folder structure, folders and files need to have meaningful names so users can have a grasp of the content without opening all folders and files. Therefore, naming conventions should be created with experimental factors encoded in them.

The original datasets we curated did not have a naming convention established during the data creation and analysis stages by the authors. For example, folders and files from different replicates or samples of the same type of experiment (e.g., same treatment and organism genotype) were often named differently, sometimes using the strain name, or the genotype name or even the treatment name. This made it much more difficult to relate samples with each other and to tell the content of the folders without the help of the data creators.

When organizing such datasets for sharing, we created naming conventions that followed relevant experimental factors and sample numbers. For example, for experiments using different genotypes and techniques in Kalita2023, we created naming conventions that had "genotype_technique_sample-number" where examples of the resulting names were: "wild_type_FISH_S1", which means cells from the wild type strain visualized using fluorescence in situ hybridization (FISH), sample 1; or "delta_hfq_pQE80L_HaloTag_S3", which means cells from the delta *hfq* mutant carrying the pQE80L plasmid visualized using HaloTag labelling, sample 3. Note also that names do not contain spaces or special characters (non-alphabetical or numerical), as this can cause errors when loading files for programmatic analysis.

Once a name convention is established, it is important to use it consistently, to ensure standardization across the dataset. Even if a researcher decides to change the file names along the project, it will be much easier to change them in an automated way (e.g., using command line or a file rename app) if the same naming convention had been consistently applied, and if similar files, e.g., files from replicate samples, are organized and saved together. Taking the previous example, if files had been named “with_cipro” and “without_cipro” and one wished to change them to “on_cipro” and “off_cipro” it would be a matter of selecting the folder where names should be changed and replace all files containing “with” in the name with “on” and all files containing “without” with “off”. However, if the researcher collecting data did not name and organized files consistently, the changes must be done by hand, which can take much longer. When possible, we used a free file-renaming software “Bulk rename utility” to rename files and folders ( https://www.bulkrenameutility.co.uk/).

The folder structure and naming conventions that we used were then documented in the main README files accompanying the dataset.

***Writing metadata: README files.*** An effective and simple way of recording and sharing metadata, especially if sharing in a generalist data repository such as Zenodo, is to add README files together with the data files ^38^. The README files allow a detailed description of the dataset without being restricted to metadata fields in the repositories. Additionally, README files are useful for keeping metadata information saved together with the files when storing and archiving data.

Examples of information to be added to README files are project title, authors (ORCID numbers), research project details (e.g., funding codes, experimental context), sample information (species, genotype, strains, etc.), experimental design, methods and parameters used, potential data reuse, dataset file/folder overview and more. When dealing with several types of data in the same dataset, such as in Kalita2023, specific README files might be added for each data type (e.g., README-FM for fluorescence microscopy data, README-SRC for source code), apart from the general README that describes the whole dataset structure (see READMEs in 17).

Furthermore, when applicable, controlled vocabularies should be used. Controlled vocabularies are formal, accessible, and widely recognized terminology for describing data and provide a list of terms to be used in a particular field. For example, MeSH (Medical Subject Headings; https://www.nlm.nih.gov/mesh/meshhome.html), a controlled vocabulary used to index biomedical information, has a list of entry terms for *Escherichia coli* and related categories. “MeSH on demand” is a tool to that automatically detects MeSH terms in a text (e.g., in an abstract) which can help to identify subject keywords. The use of controlled vocabularies to describe the data either in README files or in metadata fields in repositories will help to reduce duplications and ambiguities amongst similar datasets and will facilitate data findability and interoperability.

The process of writing README files was not done in a single step (see Figure 2). We created the READMEs and started filling them as soon as we started organizing the datasets, and gradually updated and expanded these files throughout the data curation stages. For example, information related to data organization and data cleaning were added in later stages. Researchers could therefore initiate these files at the time of data generation.

The README files were created in text format (.txt) and added to archived data on Zenodo. On GitHub, READMEs were saved in markdown format (.md).

We provide a template for READMEs with sections we believe are relevant for biological sciences datasets in our data curation materials deposited on Zenodo ^30^. An example of a real README file can be seen in Lepore2024 dataset ^28^.

### Data cleaning and transformation

After selecting the appropriate data and metadata, we verified if table files are “tidy”, i.e., well-structured consistently, and easy to manipulate and analyse. When organizing data tables for sharing, we followed practices from materials developed by our own “FAIR in (Bio)Practice” workshop ^39^ ( https://carpentries-incubator.github.io/fair-bio-practice/), and “Tidy data” ^40^.

We found that even when care has been taken when data were recorded by researchers, some mistakes or bad practices would still occur. Some of the common problems that we encountered were data tables without headers, header names that contained spaces or non-alphabetical characters, use of problematic null values, or data with misspelled names. All of these were spotted and changed before sharing.

There are different tools that can be used for data cleaning, such as *OpenRefine* ( https://openrefine.org/), or R ( https://www.r-project.org/) and Python packages specifically designed for data cleaning tasks such as pandas and Numpy ( https://pandas.pydata.org/; https://numpy.org/). We mostly used R and Python, and in some cases data cleaning was done manually. During the process of data cleaning for Scorza2022, we prepared showcase notebooks for data cleaning with R, as R markdown files ^41^. The aim of these notebooks is to provide practical training to other researchers and data curators using real data. We give examples of data cleaning and how to process the files to identify errors and fix them. For example, we show how to deal with several issues found in data tables, such as problematic header names, misspelled values, converting data to International Organization for Standardization (ISO) standards, etc.

Below we will go through some of the data cleaning steps that we have performed when sorting data tables for sharing:

***Adding / fixing header names.*** Data in tables must contain self-explanatory header names in every column that contains data. The name convention for headers can follow the one described above for folder and file names, although headers should be shorter. Practical examples of fixing/ adding header names can be found in our data cleaning notebook “data-wrangling-01.Rmd”. A python script for adding headers to a set of CSV files is also provided in our data curation materials deposited on Zenodo ^30^.

***Removing inconsistencies.*** In certain occasions when a data creator recorded new data into a spreadsheet, they either misspelled or chose a slightly different name for the same sample type or variable. This often happens when a naming convention hasn’t been established. Inconsistencies might cause problems when analysing data, especially when sorting data by sample name, as some values from misspelled / different names will be missed. For example, when sorting datasets for Scorza2022, we were dealing with 122 different wastewater treatment centres (named “sites” in the dataset), but the original data (i.e., before the data cleaning process) had 167 unique site names, indicating that the dataset probably had some site name duplications. To help spot these duplications, we sorted the sites alphabetically and found that they were mostly misspelled names. Another option for spotting these was to count the reported measurements for SARS-CoV-2 N1 gene for each site and find those that had less than 5 measurements. As each site was monitored more than few times, the ones found with less than 5 measurements would probably be the misspelled names (see “data-wrangling-02.Rmd”)

***Separating different types of values into their own individual columns.*** Sometimes authors included more than one piece of information in the same cell, which can limit data analysis. For example, when dealing with RT-qPCR data, sample information, gene analysed, and replicate number were originally all grouped in the same cell. A simple way to separate these into different columns was using “Convert Text to Columns” wizard in Excel.

***Dealing with null values.*** When presenting quantitative data, it is important to use clearly defined and consistent null indicators. Good choices for null values depending on software applications can be found here ^42^. Blank cells to indicate null values is a compatible option with most applications (R, Python, SQL) and NA is also a good option and is compatible with R. To change null values that had already been assigned, we used either a script to treat a set of files at the same time, or simply the substitution function in Excel to change one or two files. We also used a python script to delete rows containing null values, which can be found in our data curation materials on Zenodo ^30^.

***Convert data to standard formats.*** Certain types of information such as dates, time and geographical locations are often presented in different formats. For example, date elements [year(Y), month (M) and day (D)], are frequently shown as either “DD/MM/YYYY”, “DD-MM-YY”, “MM-DD-YYYY”, “DD month YYYY” and other variations. Therefore, we recommend converting all dates to ISO standard (YYYY-MM-DD or YYYYMMDD; ISO 8601:2019). The date elements can also be split into separate columns, especially when analysing data by date element. Similarly, time values and geolocations should also be converted (ISO 8601:2019 and ISO 6709:2022, respectively). We have an example of converting dates and geolocations to ISO in our notebooks “data-wrangling-02.Rmd” and “data-wrangling-05.Rmd” respectively.

***Organize Excel tables for compatibility with text formats.*** Although older Excel file formats (XLS) were originally proprietary, its current specification (XLSX) is open and can be used by third-party tools, as such it is considered open and interoperable format. However, Excel data tables should maintain their integrity when exported to text formats (CSV, TSV, TXT). Avoid creating multiple tables in the same spreadsheet, using multiple tabs, or using formatting to convey information in Excel. These practices often cause information loss and disordering of data in their proper columns and rows when exported to text format. To prevent any compatibility issues we chose to export all Excel files to CSV.

***Data transformation - converting files to open interoperable formats.*** Open file formats are non-proprietary, meaning they do need to require commercial software to be accessed, and should be used whenever possible (e.g., CSV, TIFF, MP4, XML, or FASTA). Usually, proprietary software have functionalities to export data to open formats. If not available, data producers should seek other ways to convert files whenever possible, but exceptions may apply. For example, certain proprietary formats are the standard in a particular field or certain file types might be too complicated to convert to a different format and risk losing functionality (e.g., code produced using MATLAB). Therefore, we did not modify any MATLAB code, but we did export data tables that were stored in MATLAB format to CSV.

Other examples of data cleaning and organization such as aggregating data and reorganizing columns can be found in our R notebooks ^41^. The data cleaning process such as naming headers, indicators for null values, and file formats was also recorded in the README files.

### Data and metadata sharing

***Uploading data and metadata to selected repository(ies).*** Most repositories have a web-based tool for uploading files. To deposit data on Zenodo, we saved the main data type folders in separate .zip files so users can choose specific data to download. However, for Kalita2023 the image dataset was too large, and the Zenodo platform could not handle the upload of a single large file. Therefore, we split the image dataset into the next organizational level, which was the “folder by figure” to successfully upload the files. The README files were uploaded separately so users could see the content using the preview option. It is worth noting that larger files (typically > 10GB) took longer to upload (sometimes over an hour) and the upload process was prone to fail, sometimes repeatedly (at the time of this data preparation). Even when using the University’s network this remained an issue, so it is important to consider upload time when planning the sharing of larger datasets.

Data repositories have both mandatory and optional metadata fields, and it is important to fill them as thoroughly as possible. Mandatory fields often include information such as title, authors, publication date and a description. However, optional fields are also very important for dataset findability. PIDs such as ORCID numbers, DOIs of related datasets and publications, and funding numbers integrate the dataset with other sources and research field categories and keywords maximise discoverability based on browsers search. When uploading our datasets, we filled these fields as thoroughly as possible, and linked information about code from either GitHub or GitLab.

Repositories often offer an embargo period if, for example, data submitters would like to make the data available only after a manuscript is accepted. Finally, adding a license is either mandatory or automatically assigned by the repository. Licenses will be discussed below.

***Adding an open license.*** The license will state what data users can do with the data and how or if it can be redistributed and repurposed for something else. To comply with open science and FAIR principles, an open license must be used, as it will attribute no or few restrictions on data reuse. We used CC-BY 4.0 ^43^ for the data files we shared (apart from code). CC-BY 4.0 works well for scientific data as it states that data may be used freely, but attribution must be given to the original dataset. CC0 ^44^ is a less restrictive option and does doesn’t legally require users of the data to cite the source, though attribution can still be requested as norm of good research practice. For code, the Open Source Initiative (OSI) has a list of approved licenses that comply with open source definitions. Among these, the MIT licence ^45^ is our recommended option for its simplicity, as anyone can deal with the software without restrictions. Zenodo now allows users to add more than one license to same dataset, so we could maintain files that had different license requirements together in the same upload.

***Writing a dataset publication.*** Datasets with high reusability potential can also be shared as a specific type of publication. For example, Scorza2022 was published as a “Data Descriptor” publication in *Scientific Data* ^25^. These publications describe datasets and focus on their reusability (methods for data collection and validation). Having a dataset published in a peer-reviewed journal can be an additional, valuable output derived from a properly curated dataset.

***Integrating data resources: Creating a project website.*** A more complex and longer project might benefit from having their own webpage where datasets and metadata are integrated in a user friendly and interactive way, and users can navigate the various parts of the project within the same resource. Summary paragraphs for each section and links to external resources (repositories, protocols, publications) help with finding relevant content quickly. Additionally, researchers can add information about ongoing experiments and methodologies being developed, and not be restricted to published data, attracting potential new collaborations. A webpage can also be more accessible for users that are not formal scientists or that are from different research backgrounds (e.g., citizen science, collaborators in administrative areas of the project). The main issue with project’s webpage is its durability, as web hosting involves costs and, in too many cases, the pages become inaccessible soon after their project ends. A good, free alternative is GitHub Pages ( https://pages.github.com/). They can be added to any git repository and are a cost-efficient way of providing stable web resources. We used GitHub pages to create a website for the SARS-CoV-2 RNA levels in Scotland’s wastewater project ( https://covid-ww-scotland.github.io/) where we shared not only the published datasets and publications, but also contextual information about the project, as well as links to related published protocols, ongoing research, and links of other resources such as other wastewater-based monitoring programmes.

## Discussion and perspectives

When working with data curation projects we encountered many challenges regarding data organization, and consequently issues with finding the correct data files and metadata information. In addition to this, as we worked with data from researchers that were either away in different jobs or had moved on to work on different projects, we often had to wait for their response to clarify missing or ambiguous information in their records. These factors, added to the tasks shown in Figure 2 and described above, made the data curation process take longer than anticipated. For example, for one project, creating folders and re-organizing files (excluding the process of deciding on a folder structure) took longer than 12 hours in total, and just the process of reviewing README files for completeness prior to upload took approximately 8 hours. Despite the challenges, we could still communicate with data creators and get the clarifications we needed, and the data files were all archived in safe locations that we had access to. Therefore, we were able to complete our data curation work and share data openly and as closely to FAIR principles as possible.

**Figure 4.**
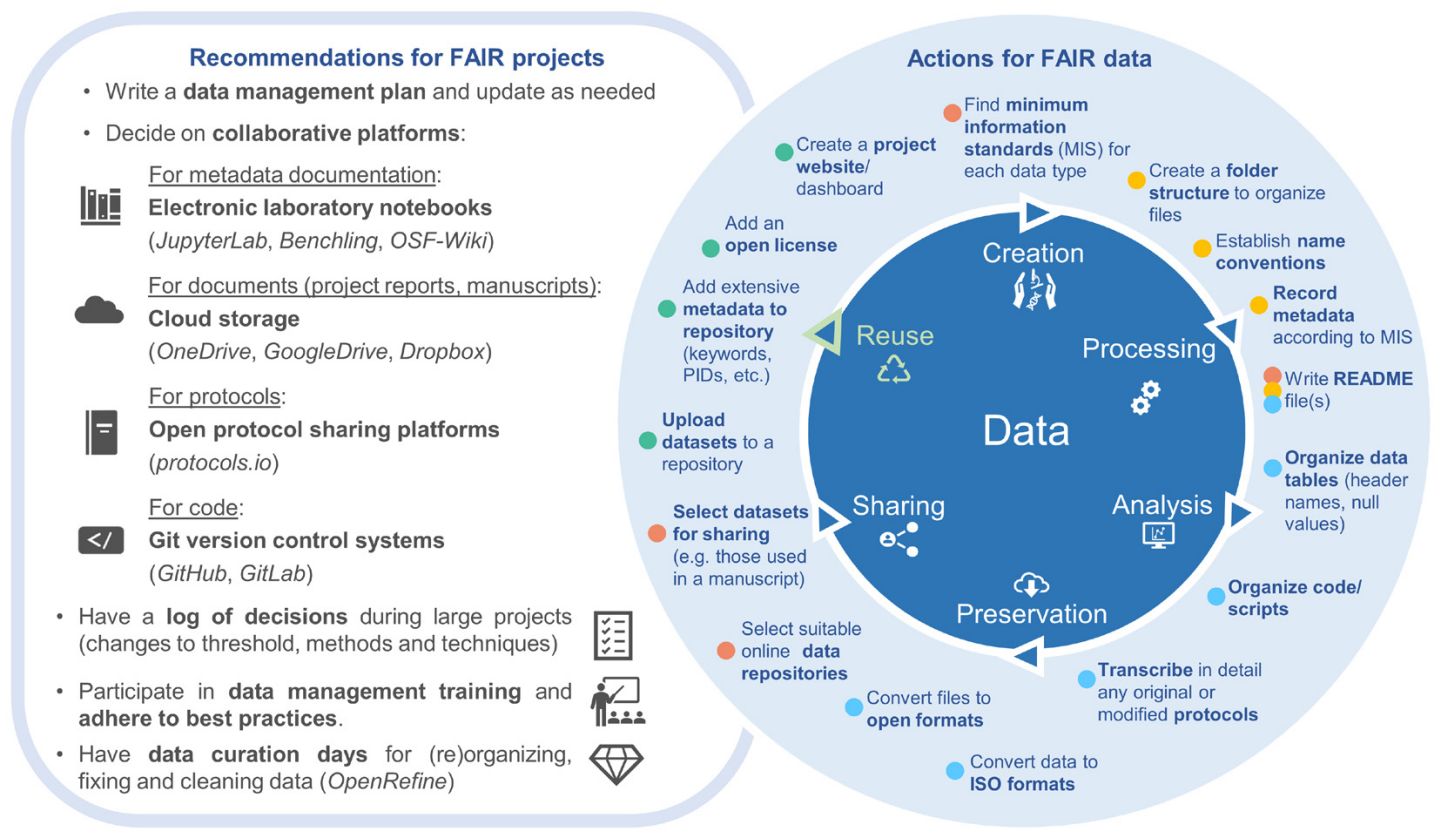
On the left are general recommendations for FAIR projects, with suggestions to assist with the actions taken to achieve FAIR data sharing. The actions are represented around the stages where they typically occur in the research data life cycle, but they can also happen repeatedly or in different stages (e.g., datasets can also be uploaded to a repository soon after data creation). Such actions were taken during the data curation process for data sharing (coloured circles next to each action relate to colours of data curation steps shown in Figure 2). If researchers incorporate these actions throughout their projects, getting data ready to share can become much quicker and more manageable.

Considering the challenges we encountered, we propose the following solutions to help to alleviate the potential burden of preparing data for sharing (most of which are also highlighted on Figure 4):

*Include README files during the data creation, processing and analysis stages:* descriptive README files should be added to data folders by researchers when creating data to help with comprehension of the datasets by themselves and future users. This will accelerate the creation of metadata for sharing.

*When preparing a manuscript, list the locations and names of data files:* identifying and finding the correct data files that should be shared can be a demanding task. Listing the location and names of data files while preparing the publication figures is a simple way to help one to quickly trace back to the raw and analysed datasets used. We suggest either a simple spreadsheet like the one on Figure 3 (also in data curation materials on Zenodo ^30^- “template_for_source_data_information.xlsx”) or a text file listing the figure numbers, a title for the data content, the location of the data files (e.g., a path in a network drive) and the file names. Naming the main contributors helps with later queries, and potentially also with the attribution of specific data deposits.

*Create naming conventions and a sensible folder structure at the beginning of the project*: The process of re-structuring the datasets, creating naming conventions and renaming files and folders took a significant amount of time during the data curation process. Ideally, naming conventions and folder structure should be established in the initial stages of the project and documented in a data management plan as well as in the README files accompanying the data, and updated as changes are made.

*Use electronic laboratory notebooks (ELNs)*: Because information from ELNs can be easily searched and reused, the use of electronic laboratory notebooks (ELNs) for record keeping was extremely helpful for us when sourcing metadata, especially when more experimental details were needed. Additionally, ELNs have a date stamp, which helped linking information from ELNs to data files that had been saved with date information, speeding up the process of gathering metadata.

*Prepare tidy tables*: Data cleaning is essential for data preservation and as it improves data quality, interoperability and reusability. Steps for data cleaning during data curation could have been minimised or even totally avoided if researchers collecting and recording data were aware of good practices for spreadsheet use and had used them during data creation, processing and analysis stages.

*Add license to code and use version control systems*: As stated previously, one of the main issues we encountered was the lack of a clear license in shared code. For code to be open it should comply with the open-source definition (see https://opensource.org/osd/), where the source-code is included and shared using an open-source license. Another problem when reusing code from others, was broken links when trying to download a software package. Therefore, we advise researchers to use a version control system such as Git and share it on *GitHub* ( https://github.com/) or *GitLab* ( https://gitlab.com/) to share their code. If possible, we recommend doing this from the beginning of the project, as it will also allow collaborative work and the version control will help code developers to keep track of the changes without losing information or breaking the code. Features such as sharing ownership of repositories only with collaborators and restricting visibility might help researchers feel more confident before sharing all the code publicly.

*Incorporate good data management practices during the research data life cycle*: If datasets are left to be organized only at the end of the projects, complying with FAIR principles can be daunting. In cases where research groups do not have the resources to assign a data curator for a project, and the data creators are not careful when recording meta(data), sharing datasets can even become impossible, or if shared, the data are unlikely to be reusable. Figure 5 summarizes some of the important data management decisions to make before starting a FAIR project. Researchers should also be familiar with best practices in data management and data file organization, for example by attending specific training. Tips for implementing new research practices after such training were recently published ^46^. We also prepared a list where we suggest online resources for self-paced reading or training in Table 1. Good data management practices are not only important for the sharing stage of the dataset, but also for the continuation of the projects. A common factor in academic research environments is that researchers, especially those at post-graduate and postdoctoral levels, often have short stays in laboratories, leaving research projects to be continued by new group members. Good data management will help new group members to access, find and understand previously recorded data and all the underlying experimental details. Having all-hands “data curation days” for (re)organizing, fixing and cleaning data can be a good option to keep datasets organized during a project.

**Table 1.** List of suggested training materials for Open and FAIR data.

Course / Resource name	Link to resource	Source (citation)
FAIR in (biological) Practice	https://carpentries-incubator.github.io/fair-bio-practice/	39
The Turing Way guide for reproducible research	https://book.the-turing-way.org/reproducible-research/ reproducible-research/	47
Good enough practices in scientific computing	https://carpentries-lab.github.io/good-enough-practices/	37, 48
Escape from spreadsheet hell	https://instr.iastate.libguides.com/spreadsheets/home	49
Library Carpentry: Tidy data for Librarians	https://librarycarpentry.org/lc-spreadsheets/	50
Version control with Git	https://swcarpentry.github.io/git-novice/	51
Data cleaning with OpenRefine for Ecologists	https://datacarpentry.org/OpenRefine-ecology-lesson/	52
ELIXIR guidelines and best practices	https://elixir-europe.org/what-we-offer/guidelines https://faircookbook.elixir-europe.org/ https://rdmkit.elixir-europe.org/	ELIXIR Europe ( https:// elixir-europe.org/)
Bringing data to life: Data management for the biomolecular sciences	https://www.ebi.ac.uk/training/online/courses/bringing- data-to-life-data-management/	53

*Use collaborative digital resources*: Research projects that involve several collaborators must agree on an organizational structure that is meaningful for all members to work effectively, avoiding time wasting associated with file retrieval and preventing file duplications and data loss. To assist with (meta)data co-creation in a developing project, we recommend the use of cloud storage services ( *OneDrive*, *GoogleDrive*, *Dropbox*) when working on documents such as data management plans, project reports, and manuscripts ( Figure 4). There is also a selection of electronic laboratory notebooks that can be used jointly by different members of a project, such as *Benchling* (paid, but has free version https://www.benchling.com/) *, OSF Wiki* (free; https://osf.io/pfhqc/wiki/home/) or *JupyterLab* for users who perform data analysis using programming in Python, R and others (free; https://jupyter.org/).

*Share protocols*: Using open online protocol platforms can help to minimize confusion with different versions of protocols and lack of detailed information. Also, platforms such as *protocols.io* issue a DOI for published protocols, which adds extra research outputs to a researcher’s portfolio. In some cases, depending on its originality and impact, protocols can also be shared as separate peer-reviewed publications (e.g., in *Nature Protocols*, *Lab Protocols* -PLOS, *Methods and Protocols* -MDPI, or *JoVE Journal*).

In Supplementary Figure 1 (see in our data curation materials on Zenodo ^30^) we show how sharing a dataset in a digital data repository such as Zenodo will help to comply with all elements of FAIR data.

Sharing open and FAIR data is not only beneficial for the scientific community and public in general due to the availability of reusable datasets, but it is also a benefit for the data owners who are making their data available. For example, evidence shows that linking shared datasets to papers increases citations ^54, 55^. Additionally, sharing datasets as separate outputs provides the opportunity to vary authorship and give proper credit to different contributors. For example, the first author of a deposited dataset might be its lead producer, who is not the first author of the overall publication.

Finally, following the adoption of the San Francisco Declaration on Research Assessment (DORA) by most research institutions and funders, the way that researchers' contributions are being evaluated is moving beyond traditional journal indicators and considering wider contributions, often using Narrative CVs. As an example, a narrative CV template proposed by UKRI (The Résumé for Research and Innovation R4RI) suggests including a researcher’s contributions “to the development of new tools, methodologies”, “to the wider research and innovation community”, and “to broader research or innovation-users and audiences, and towards wider societal benefit”. The research outputs shared as datasets in repositories, online protocols, open collaborative code on GitHub, project webpages, and dataset publications will all count positively in this new evaluation system and will boost a researchers’ impact helping them to stand out from the crowd.

## Ethics and consent

Ethics approval and consent were not required

## Data Availability

Zenodo: Data curation materials in "Daily life in the Open Biologist's second job, as a Data Curator". https://zenodo.org/doi/10.5281/zenodo.12734112 ^56^. The project contains the following data: The python scripts we used for data cleaning The template for source data information The README template Supplementary Figure 1 Data are available under the terms of the Creative Commons Zero "No rights reserved" data waiver (CC0 1.0 Public domain dedication).

## Software availability

The scripts used for data curation are shared in our notebooks on GitHub (BioRDM data wrangling with R: Showcase notebooks for data cleaning with R, https://github.com/BioRDM/data-wrangling-with-r) and on Zenodo (Data curation materials in "Daily life in the Open Biologist's second job, as a Data Curator”, https://zenodo.org/doi/10.5281/zenodo.12734112).
