# Supplementary material for: Daily life in the Open Biologist’s second job, as a Data Curator
Source: Wellcome Open Res. 2024 Dec 5;9:523. Originally published 2024 Sep 12. [Version 2] doi: 10.12688/wellcomeopenres.22899.2 (PMC11445645; doi:10.12688/wellcomeopenres.22899.2)
Supplement: Peer review history

# Reviewer report: Reviewer response for version 2

**DOI:** 10.21956/wellcomeopenres.25748.r114691

**Published:** 2024-12-06

**Authors:** Sharif Islam

**Affiliations:** 1 Naturalis Biodiversity Center, Leiden, South Holland, The Netherlands

no new comments.

Is the work clearly and accurately presented and does it cite the current literature?

Yes

If applicable, is the statistical analysis and its interpretation appropriate?

Not applicable

Are all the source data underlying the results available to ensure full reproducibility?

Yes

Is the study design appropriate and is the work technically sound?

Yes

Are the conclusions drawn adequately supported by the results?

Yes

Are sufficient details of methods and analysis provided to allow replication by others?

Yes

Reviewer Expertise:

Biodiversity Informatics, Data Architecture, Data Standards, Research Data Management, FAIR implementation.

I confirm that I have read this submission and believe that I have an appropriate level of expertise to confirm that it is of an acceptable scientific standard.

# Reviewer report: Reviewer response for version 2

**DOI:** 10.21956/wellcomeopenres.25748.r114692

**Published:** 2024-12-06

**Authors:** Yojana Gadiya

**Affiliations:** 1 Fraunhofer Institute for Translational Medicine and Pharmacology, Hamburg, Germany

All comments seem to be addressed.

Is the work clearly and accurately presented and does it cite the current literature?

Yes

If applicable, is the statistical analysis and its interpretation appropriate?

Yes

Are all the source data underlying the results available to ensure full reproducibility?

Yes

Is the study design appropriate and is the work technically sound?

Yes

Are the conclusions drawn adequately supported by the results?

Yes

Are sufficient details of methods and analysis provided to allow replication by others?

Yes

Reviewer Expertise:

Chemoinformatics, FAIRification, Data science

I confirm that I have read this submission and believe that I have an appropriate level of expertise to confirm that it is of an acceptable scientific standard.

# Reviewer report: Reviewer response for version 1

**DOI:** 10.21956/wellcomeopenres.25215.r99252

**Published:** 2024-10-15

**Authors:** Jian Qin

**Affiliations:** 1 Syracuse University, Syracuse, New York, USA

This paper provides a detailed account of curation planning, decision-making with justifications, and execution of the plan for three datasets in biological/biomedical research. The strength of this paper is in its very practical, step-by-step documentation of how curation decisions were made and executed, which offers insightful guidance and reference for data curators. The technical details involving use of cloud services, uploading large files, and creating README documents are especially valuable as these are the foundational conditions for ensuring the FAIR-ness of data.

 The paper is very easy to read and follow. The narrative follows the workflows of data curation with frequent reference to the visual steps (in figures) enforced the clarity and coherence. 

 One thing I had hoped to see but the paper only mentioned as a sideline in this paper is the challenges and difficulties related data culture and practice. I was curious about what perceptions and reactions the scientists had for an "outsider" data curator (non-project member) to have access to and "messing around" with their data, what attitudes they had for the various "imposed" best practices (such as data formats, cleaning, naming conventions, etc.), and how the data curation team handled such challenges and hurdles. If a formal evaluation is not feasible (I understand this paper has covered a great deal) in this paper, some informal assessment and even anecdotes for scientists' feelings toward this data curation process would have provided a richer story for this data curation case analysis. After all, technical operations are usually the easier part in data curation; it is the human and culture side of the data curation that is more difficult and challenging because changes in human nature and culture take more time and effort to happen. 

Is the work clearly and accurately presented and does it cite the current literature?

Yes

If applicable, is the statistical analysis and its interpretation appropriate?

Not applicable

Are all the source data underlying the results available to ensure full reproducibility?

Yes

Is the study design appropriate and is the work technically sound?

Yes

Are the conclusions drawn adequately supported by the results?

Yes

Are sufficient details of methods and analysis provided to allow replication by others?

Yes

Reviewer Expertise:

Metadata, ontology, data curation/research data management, scientific collaboration networks, science of science

I confirm that I have read this submission and believe that I have an appropriate level of expertise to confirm that it is of an acceptable scientific standard.

# Response

**Published:** 2024-12-02

**Authors:** Livia Scorza

**Affiliations:** School of Biological Sciences, The University of Edinburgh, Edinburgh, Scotland, UK

Thank you for your feedback. The researchers we worked with were generally receptive to our data curation approach as they understood the value of shared FAIR data. Additionally, because the release of datasets was a requirement from the journal, the data creators recognized the value of having such support during manuscript preparation, which can be very time consuming. We added a new paragraph at the end of the manuscript to comment on our collaboration experience and their perception. “ *We believe our data curation work provided new perspectives to the data creators we worked with, helping them recognize the tangible benefits of sharing research data. They were not only receptive to our data curation and sharing approach but also valued our taking on the time-intensive preparation of FAIR data, particularly when undertaken alongside manuscript preparation. Formal co-authorship did not follow in publications but this positive, personal engagement highlights one bottom-up approach to change research culture towards openness and FAIR data sharing*” 

# Reviewer report: Reviewer response for version 1

**DOI:** 10.21956/wellcomeopenres.25215.r99247

**Published:** 2024-10-01

**Authors:** Sharif Islam

**Affiliations:** 1 Naturalis Biodiversity Center, Leiden, South Holland, The Netherlands

The article discusses practical steps for data curation, highlighting the challenges and solutions involved in making biological data FAIR. The paper is well-organised, with a logical flow of topics. The figures are helpful, providing clear representations of processes such as describing the data life cycle steps and the template for a spreadsheet.

 The examples, particularly those on establishing naming conventions for files and folders, decision logging, README guidelines, file reorganisation, and communication with researchers, are based on actual projects, adding significant practical value. The mention of the time required for some of these tasks ( *"... for one project, creating folders and reorganizing files (excluding the process of deciding on a folder structure) took longer than 12 hours in total"*) reflects the day-to-day reality of these efforts, which are often not visible and reported in the final publication. 

 Although software and GitHub are briefly mentioned, the paper would benefit from integrating FAIR principles for research software (https://doi.org/10.1038/s41597-022-01710-x), particularly the idea of software citation practices (https://www.software.ac.uk/publication/how-cite-and-describe-software). As FAIR principles are increasingly extended to software, algorithms, and protocols, it is important to address how these digital objects can be managed alongside datasets.

 Another useful addition to the paper would be the discussion of collaboration and communication with data stewards and IT support personnel within organisations. The paper touches on large file uploads to repositories like Zenodo, which can be problematic with files larger than 10GB. Early involvement of IT personnel in such cases could prevent issues like slow uploads or failures. Many institutions lack the necessary resources, which underscores the importance of early communication between data creators and IT departments to ensure smooth submissions.

 While the paper suggests attending data curation workshops, it would be helpful to emphasise the organisational challenges of securing time, funding, and management support. Many institutions still mistakenly associate FAIR and open datasets as "cost-free", which can lead to an underestimation of the resources needed for proper curation.

 The paper could also highlight the importance of raising FAIR awareness at the institutional level, specifically pointing out how researchers need support to justify the time and resources required for data curation. This ties into the discussion around the shift to Narrative CVs, which value contributions beyond publications.

Is the work clearly and accurately presented and does it cite the current literature?

Yes

If applicable, is the statistical analysis and its interpretation appropriate?

Not applicable

Are all the source data underlying the results available to ensure full reproducibility?

Yes

Is the study design appropriate and is the work technically sound?

Yes

Are the conclusions drawn adequately supported by the results?

Yes

Are sufficient details of methods and analysis provided to allow replication by others?

Yes

Reviewer Expertise:

Biodiversity Informatics, Data Architecture, Data Standards, Research Data Management, FAIR implementation.

I confirm that I have read this submission and believe that I have an appropriate level of expertise to confirm that it is of an acceptable scientific standard.

# Response

**Published:** 2024-12-02

**Authors:** Livia Scorza

**Affiliations:** School of Biological Sciences, The University of Edinburgh, Edinburgh, Scotland, UK

Thank you for your suggestions. We have included the suggested references related to sharing software, modifying slightly our recommendations on software/ code:  

*“Therefore, we advise researchers to follow good practices when describing and citing software (https://www.software.ac.uk/publication/how-cite-and-describe-software). Additionally, researchers must use a version control system such as Git and share it on GitHub ( https://github.com/) or GitLab ( https://gitlab.com/). If possible, we recommend using Git from the beginning of the project, as it will allow collaborative work and the version control will help code developers to keep track of the changes without losing information or breaking the code. Features such as sharing ownership of repositories only with collaborators and restricting visibility might help researchers feel more confident before sharing all the code publicly. Further guidance on implementing FAIR principles specifically for research software can be found in the published work from The FAIR for Research Software Working Group (Barker, M., Chue Hong, N.P., Katz, D.S. et al. Introducing the FAIR Principles for research software. Sci Data 9, 622 (2022).* *https://doi.org/10.1038/s41597-022-01710-x* *)”*

 We also included a paragraph in the recommendations (Discussion) commenting on the need of integrating different digital objects:

 “ *Integrate resources and digital objects: related digital objects (datasets, protocols, software, etc. that were uploaded separately in repositories) can be integrated in a dedicated project website as discussed previously. However, it is also important to interlink all related data outputs by adding the corresponding URLs or DOIs to these objects in the appropriate metadata fields and/or README files, linking the data that is stored across different platforms. This laborious, manual process will hopefully be alleviated by better resource integration in future.*” We also included a paragraph commenting that researchers must cost data management and curation services in grant applications, when those are required: “ *Our recommendations aim to help experimental researchers to incorporate FAIR principles to their data in their daily work. However, it is important to highlight that many research projects present a complexity that will require professional data management advice and data curation services. These must be identified and costed when securing funding during grant application. Researchers should also explore the data management services available from their institution and cost for those when necessary (e.g., project and data management platforms, data sharing and archiving, computational services, etc.)*”

# Reviewer report: Reviewer response for version 1

**DOI:** 10.21956/wellcomeopenres.25215.r99253

**Published:** 2024-09-30

**Authors:** Yojana Gadiya

**Affiliations:** 1 Fraunhofer Institute for Translational Medicine and Pharmacology, Hamburg, Germany

The article sheds light on the importance of FAIR. It is known in the research community that data should be FAIR, but researchers struggle with the applicability of these principles in real-world scenarios. Additionally, the technical jargon for biologists adds to the reluctance to "do FAIR." This article is a great approach to bridging the gap between biologists and other researchers and the FAIR principles.

 A few suggestions are:

 1.To change reference no. 12 to https://www.nature.com/articles/s41587-019-0080-8 since sharing is superseded by fair sharing now.

 2. For the " *“data availability* *statement” *statement in the introduction, I would add that despite this effort, there is no strict regulation of content entered here, and statements like "data will be provided on request" have also emerged recently.

 3. When describing about the three use cases discussed, I would recommend adding a few lines on the different data formats that you would be dealing with as well as different software where the data was scattered in.

Is the work clearly and accurately presented and does it cite the current literature?

Yes

If applicable, is the statistical analysis and its interpretation appropriate?

Yes

Are all the source data underlying the results available to ensure full reproducibility?

Yes

Is the study design appropriate and is the work technically sound?

Yes

Are the conclusions drawn adequately supported by the results?

Yes

Are sufficient details of methods and analysis provided to allow replication by others?

Yes

Reviewer Expertise:

Chemoinformatics, FAIRification, Data science

I confirm that I have read this submission and believe that I have an appropriate level of expertise to confirm that it is of an acceptable scientific standard.

# Response

**Published:** 2024-12-02

**Authors:** Livia Scorza

**Affiliations:** School of Biological Sciences, The University of Edinburgh, Edinburgh, Scotland, UK

Thank you for the suggestions. The Reference 12 is within a set of references that points to standards in Biology (4-12). The FAIRsharing publication suggested is referenced in number 3. We also added the following sentence in the first paragraph about data availability statements:   “Additionally, many peer-reviewed journals now have data sharing policies that require the inclusion of a “data availability statement” where authors must add the links to repository records that hold the datasets supporting the results presented *(although often there is no strict requirement on the content added in this section where data deposition is enforced, and researchers can add sentences such as “data will be provided on request”)*.

# Reviewer report: Reviewer response for version 1

**DOI:** 10.21956/wellcomeopenres.25215.r99244

**Published:** 2024-09-18

**Authors:** Karim Fouad

**Affiliations:** 1 Faculty of Rehabilitation Medicine, University of Alberta, Edmonton, Alberta, T6G 2G4, Canada

This is a very timely article focusing on FAIR data sharing and related challenges. The current cultural change in publishing research findings is a torturous path, with many researchers feeling the burden of new mandates. This manuscript provides important guidance and a good overview of the challenge. There is only one major shortcoming as this manuscript seems to focus on data associated with published papers. These papers are generally positive and promote a well-recognized publication bias. This could be counteracted by publishing all research data, an important part of the NIH data management and sharing mandate (which by the way should be mentioned in this manuscript). As it reads the authors do not mention the publication of data that are frequently referred to as negative. It seems that this is a missed opportunity.

**Minor comments:**

- it would be useful if the authors discuss the use of Common Data Elements in the section regarding "header naming convention” or “controlled vocabularies”.  CDEs are associated with and promoted by the NIH also in preclinical research, and might become globally accepted.
- Considering that researchers struggle with data terminology, a better definition of meta data would be useful, including the introduction of the term data dictionaries
- many general repositories consider themselves as FAIR, however it is difficult to define and control for fairness, especially the reusability and thus many do underdeliver in that regard. This challenge could be discussed

Is the work clearly and accurately presented and does it cite the current literature?

Partly

If applicable, is the statistical analysis and its interpretation appropriate?

Not applicable

Are all the source data underlying the results available to ensure full reproducibility?

No source data required

Is the study design appropriate and is the work technically sound?

Yes

Are the conclusions drawn adequately supported by the results?

Yes

Are sufficient details of methods and analysis provided to allow replication by others?

Yes

Reviewer Expertise:

As a preclincial researcher I have started a FAIR share data repository, we provide curation and regularly publish advice on data management: e.g.: doi: 10.1016/j.expneurol.2024.114815

I confirm that I have read this submission and believe that I have an appropriate level of expertise to confirm that it is of an acceptable scientific standard, however I have significant reservations, as outlined above.

# Response

**Published:** 2024-12-02

**Authors:** Livia Scorza

**Affiliations:** School of Biological Sciences, The University of Edinburgh, Edinburgh, Scotland, UK

Thank you for the suggestions and the feedback. We have added NIH in the funding bodies requirement examples: " *Data sharing is not only good research practice, but it has become a requirement for many datasets funded by most funding bodies (e.g., UKRI, Wellcome, Royal Society, EMBO, NIH).*"

**Unpublished results/ Negative data**: In the paper we focused on research data associated with publications because our collaboration projects were specifically on preparing data for that purpose. However, we agree we could have commented that the recommendations proposed in the discussion are applicable to all data, including negative data, as part of good research practice. Therefore, we added the following paragraph to the discussion: " *While the data curation described in this paper focuses on published datasets, the underlying principles can (and should) be extended to encompass all associated data, including so-called "negative data"—data that does not support the initial hypothesis. Although some journals are now dedicated to or open to publishing negative data, researchers can still share these datasets in repositories like Zenodo, adhering to FAIR principles, and publicise the shared data by means other than a journal publication*"

**CDEs and data dictionaries**: the paper highlights our experience curating data in biology and focuses on recommendations based on problems we encountered along the way. Although we recognize the value of CDEs and data dictionaries, we do not mention them as they were not part of our practical work (for example, CDEs are more often used in clinical research and environmental science, rather than the data context shown in the paper).

**Metadata**: we added a couple of sentences to improve its definition in the text: In the Introduction: “(…i.e., datasets are assigned with persistent identifiers such as a digital object identifier (DOI), indexed in a searchable resource and described with rich metadata. *(metadata can be defined as the records that describe and provide information about the data)*. In the Methodology “Metadata. Metadata is the description of the data, and it is particularly important for data reproducibility. *Good metadata must provide all information about the dataset content, such as authors, context of research, samples, protocols, data formats and file organization, etc*”. **F**

**AIR and repositories**: Along the paper we make clear that only depositing research data in repositories does not make data FAIR. Data needs to be properly described, transformed and organized to qualify as suitable for FAIR sharing. To highlight that in the discussion we added the following: “In Supplementary Figure 1 (see in our data curation materials on Zenodo) we show how sharing a dataset in a digital data repository such as Zenodo will help to comply with all elements of FAIR data ( *so long as data is properly described and organized following FAIR principles, as many repositories do not enforce extensive metadata or interoperable formats*)”.
